# The incidence of gestational diabetes mellitus among women with polycystic ovary syndrome: a meta-analysis of longitudinal studies

**DOI:** 10.1186/s12884-022-04690-3

**Published:** 2022-04-29

**Authors:** Qingzi Yan, Dan Qiu, Xiang Liu, Qichang Xing, Renzhu Liu, Yixiang Hu

**Affiliations:** 1 Department of Pharmacy, Xiangtan Central Hospital, Hunan, China; 2 Department of Social Medicine and Health Management, Xiangya School of Public Health, Central South University, Changsha, Hunan China

**Keywords:** Gestational diabetes mellitus, Polycystic ovary syndrome, Incidence, Meta-analysis, Longitudinal study

## Abstract

**Background:**

Previous studies have shown that polycystic ovary syndrome is a predictor of gestational diabetes mellitus, but we do not know exactly how many polycystic ovary syndrome patients may develop gestational diabetes mellitus. Currently, the incidence of gestational diabetes mellitus among women with polycystic ovary syndrome varies greatly across studies, ranged from 4.12% to 59.50%. Besides, many factors have been found to be related to the incidence of gestational diabetes mellitus among women with polycystic ovary syndrome, but the results among different studies are not consistent. The possible causes of inconsistencies between the current estimates were unclear. This review aimed at exploring the pooled incidence of gestational diabetes mellitus among women with polycystic ovary syndrome, summarizing possible causes of the inconsistencies in the current estimates, try to provide a reference for prevention of gestational diabetes mellitus and polycystic ovary syndrome in the future.

**Methods:**

Systematic searches of different databases (including EMBASE, Web of Science, MEDLINE, The Cochrane Library, CNKI and PubMed) were conducted for studies published until 31 May 2021. Statistical analyses were performed using R software, the pooled incidence of gestational diabetes mellitus among polycystic ovary syndrome patients was combined using random effects model. Cochrane’s “Tool to Assess Risk of Bias in Cohort Studies” was used for quality assessment.

**Results:**

Twenty-two longitudinal studies were included. A total of 24,574 women with polycystic ovary syndrome were identified in the 22 articles, of which 4478 were reported with gestational diabetes mellitus. The pooled incidence of gestational diabetes mellitus among women with polycystic ovary syndrome was 20.64%, with a 95% CI of 14.64% to 28.30%. In the meta-regression model, several variables including age, area, quality score and sample size were suggested as significant sources of heterogeneity, accounted for 77.57% of the heterogeneity across studies.

**Conclusions:**

Evidence in this review suggests that gestational diabetes mellitus were common among women with polycystic ovary syndrome. More research is needed to found effective interventions for preventing gestational diabetes mellitus among women with polycystic ovary syndrome.

**Supplementary Information:**

The online version contains supplementary material available at 10.1186/s12884-022-04690-3.

## Introduction

The incidence of diabetes mellitus (DM) is increasing across the world. This is also the case for diabetes in pregnancy women – gestational diabetes mellitus (GDM) [1]. In contrast to overt diabetes mellitus, gestational diabetes mellitus is defined as any degree of glucose intolerance with onset or first recognition during pregnancy, perhaps from exaggerated physiologic changes in glucose metabolism [2, 3]. The available evidence suggests that GDM was associated with adverse outcomes for mothers and offspring in the short or long term [4–6]. Also, evidence indicated that GDM is one of the leading causes of morbidity and mortality for both mothers and infants worldwide [7]. There are a range of epidemiological studies showing that gestational diabetes mellitus is quite prevalent over the world [7–10], the prevalence ranged from 5.40% to 14.80%.

As one of the most common endocrine disorder affecting women during the reproductive years, polycystic ovary syndrome (PCOS) is a syndrome of ovarian dysfunction characterized by chronic anovulation, hyperandrogenism, and typical morphologic changes of the ovaries based on ultrasonographic examination [11–13]. The prevalence of PCOS is estimated to be 5.00%-14.00% among women during the reproductive years [14–16], and affected patients often present with symptoms and signs of menstrual irregularity, obesity and infertility [2]. Previous studies have indicated that women with PCOS are at an increased risk of developing GDM [17–22]. When women developed with both PCOS and GDM, they may be at a higher risk of developing adverse pregnancy outcomes [23]. It is said that women with both PCOS and GDM have a higher risk of developing pregnancy induced hypertension and preeclampsia and of delivering preterm [24]. Furthermore, newborns of women with both PCOS and GDM may have an increased risk of developing neonatal hyperbilirubinemia [24], metabolic and cardiovascular diseases [25].

Although previous studies have shown that PCOS is a predictor of GDM, we do not know exactly how many PCOS patients may develop GDM. Currently, the incidence of GDM among women with PCOS varies greatly across studies, ranged from 4.12% to 59.50% [26, 27]. Besides, a lot of factors have been founded to be associated with the incidence of GDM among women with PCOS, such as age, overweight, obesity and smoking, but the results were not consistent between different researches [28–31]. The possible causes of the inconsistencies among current studies are unclear. In order to take effective measures to reduce the negative consequences caused by GDM and PCOS, there is a need for more accurate estimates of the incidence of GDM among women with PCOS, and to found the possible causes of the inconsistencies among the current studies. This study aimed at exploring the pooled incidence of GDM among women with PCOS, summarizing possible causes of the inconsistencies in the current estimates, try to provide a reference for prevention of GDM and PCOS in the future.

## Methods

This review was reported in accordance with the PRISMA guideline and Meta-analyses Of Observational Studies in Epidemiology (MOOSE) guidelines [32, 33]. See Supplementary data (Table S5 and Table S6) for the details.

### Search strategy

Chinese National Knowledge Infrastructure (CNKI), PubMed, the Cochrane Library, MEDLINE, EMBASE, Web of Science, were independently searched for published articles by two reviewers (QZY and DQ), with no restrictions on language or date of publication up until 31 May 2021. The following search terms were used: ‘Polycystic Ovary Syndrome’ (including ‘Polycystic Ovary Syndrome’, ‘PCOS’, ‘polycystic ovarian syndrome’, ‘polycystic ovary disease’, ‘Ovarian Cysts’, ‘Stein Leventhal Syndrome’, and ‘poly cystic ovarian syndrome’.); “gestational diabetes mellitus” (including ‘gestational diabetes mellitus’, ‘gestational diabetes’, ‘GDM’, ‘gestational’, ‘insulin dependent diabetes’, ‘non-insulin dependent diabetes’ and ‘pregnancy-induced diabetes’); Longitudinal study (including ‘longitudinal study’, ‘longitudinal Survey’, ‘follow up study’, ‘cohort study’, ‘epidemiologic Studies’ and ‘observational study’). See Table S1 for the search strategy.

### Eligibility criteria

If the studies meet the following criteria, they were included: (1) the study was longitudinal observational study; (2) the participants were woman with polycystic ovary syndrome; (3) information about incidence of gestational diabetes mellitus among women with polycystic ovary syndrome was provided; (4) the full article was written in Chinese or English. If the studies were meta-analysis, review, conference abstract, comments, or protocol, they were excluded.

### Data extraction

The data extraction was conducted independently. By using standardized data extraction form, two reviewers (QZY and DQ) checked the titles of the search records, abstracts and full-texts of the initial search records independently with Endnote software (Endnote X9 were used during the data extraction). Data were extracted on country or area, the first author, publication year, sample size, mean age, mean BMI, percentage of overweight/obese patients, percentage of primigravida, percentage of smokers, tools used to identify GDM, incidence of GDM, and quality score of these included studies. Any discrepancies that emerged in the procedures were resolved by involving a third author (XL).

### Quality assessment

Two reviewers (RZL and YXH) used the Cochrane’s “Tool to Assess Risk of Bias in Cohort Studies”, evaluated the methodological quality of these included studies, which has been widely used to evaluate the quality of observational studies [34].

The included researches were scored according to eight criteria, such as assessment of exposure, selection of exposed and non-exposed groups, the present of outcome of interest, adjustment of the confounding variables, assessment of possible confounding factors, assessment of outcomes, and follow-up of the research. The included studies were evaluated in relation to eight question using a 4-Likert scale, including “definitely no”, “probably/mostly no”, “definitely yes”, and “probably/mostly yes”. The quality of a study was considered high/acceptable if all domains were evaluated favorably (ie, “definitely yes” or “probably/mostly yes”).

### Statistical analyses

When data were available for 3 or more studies, incidence of gestational diabetes mellitus was combined [35]. Quantitative subgroup analysis was performed when 4 or more studies were available [36]. All statistical analyses in this study were performed using the “meta” (4.13–0) and “metafor” package (2.4–0) of R version 4.0.0 [37]. Heterogeneity between the included studies was evaluated by the Cochran's Q test and quantified by I^2^ statistic [37, 38]. When the results of I^2^ greater than 50%, means moderate heterogeneity, and greater than 70% means high heterogeneity [36]. As the authors expected considerable heterogeneity, pooled incidence of gestational diabetes mellitus was calculated with the random effects model [39]. Based on random effects model (the DerSimonian and Laird method), the pooled incidence of GDM among women with PCOS was combined using Logit transformation method in the current study [38]. In order to compare the incidence of GDM from different studies, subgroup analysis was conducted. Previous research indicated that subgroup analyses and meta regression should be interpreted with caution [39], this study planned a priori to limit the subgroup analyses to a limited number of background characteristics, including area, mean age, mean body mass index (BMI), percentage of overweight/obese patients, percentage of primigravida, percentage of smoking patients, sample size, and quality score (see Table S4 for the details). The difference between those subgroups was evaluated using the Cochran's Q chi-square tests [37, 38]. The general linear (mixed-effects) meta-regression models were performed to explore potential moderators on the heterogeneity[37]. Freeman-Tukey double arcsine method were used when the meta-regression analyses were conducted. Publication bias was investigated by funnel plot and Egger's test [38]. To evaluate the consistency of these results, sensitivity analysis was performed by excluding studies one by one [38]. All the statistical tests were 2-sided, with a significance threshold of *P* < 0.05.

## Results

### Result of literature search

**Fig. 1:**
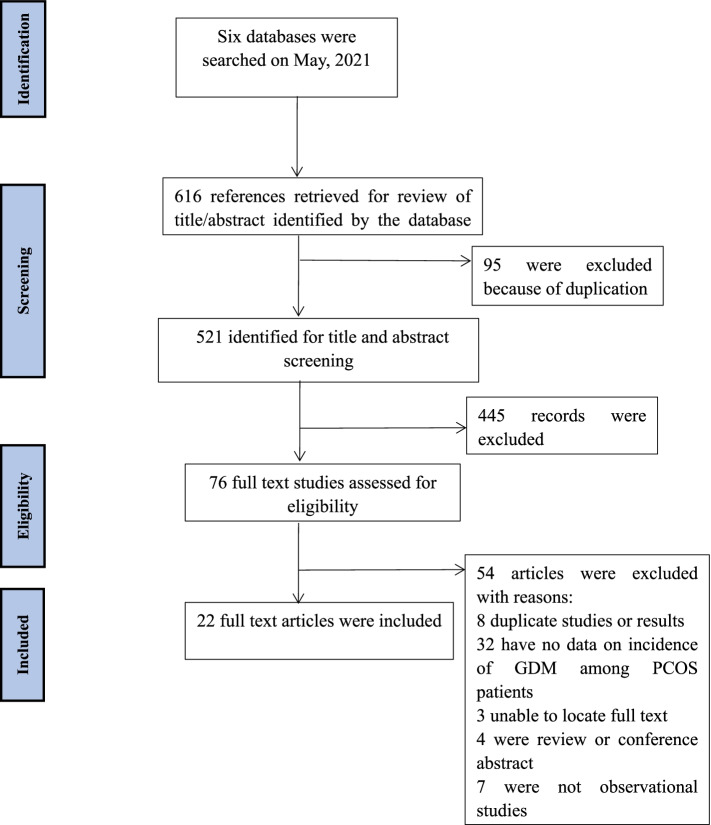
Flow of studies through review

As reported in Fig. 1, 616 studies were identified. In those studies, 95 duplicates were excluded. By screening the titles and abstracts, 445 irrelevant studies were excluded. Based on the selection criteria, 76 potentially relevant full-text papers were assessed. Further, due to the following reasons: have no data on incidence of GDM among women with PCOS (*n* = 32); duplicate articles or results (*n* = 8); not observational study (*n* = 7); review or conference abstract (*n* = 4); unable to locate full text (*n* = 3), 54 studies were excluded. Finally, 22 eligible studies were included in this review. The reliability for the full-text review between the two reviewers (QZY and DQ) was rated as good (Kappa = 0.76) [40].

### Characteristics of the included studies

The characteristics of the 22 included studies [1, 26, 27, 41–59] were presented in Table 1. Eighteen of them were reported in English and 4 were reported in Chinese. Most of the included studies were from European and Asia, such as Finland and China. From the 22 studies, 9 (40.91%) studies were rated as high or acceptable quality and 13 (59.09%) were rated as low quality. Specifically, most studies have clear inclusion criteria for the patients and most of them used reliable assessment criteria to diagnose GDM. However, a few of the included studies failed to clarify whether baseline GDM patients were excluded from incidence calculations. In addition, 59.09% (13/22) of the included studies with a sample size ≤ 300. Details of the quality assessments for the 22 included papers are reported in Table S2 and Table S3.

**Table 1:** study characteristics of included studies

First author	Country/Area	Event/N	Study characteristics	Quality score
Sammeli West et al. (2020) [42]	Finland	25/197	Survey time:/ Mean age: 31.0 Mean BMI: 25.2 Diagnostic criteria: 2-h 75-g OGTT; at 24–28 weeks	Low
Mahnaz Bahri Khomami et al. (2019) [54]	Australia	34/354	Survey time: 2004–2011 Mean age: 30.3 ± 4.5 Mean BMI: 26.1 ± 5.8; percentage of overweight/obesity patients: (177/354) SEI: 46.3 ± 13.8 Smoking: 5.9% Drinking: 6.8% Diagnostic criteria: WHO criteria (2013)	Low
J-Z Chen et al (2016) [52]	China	208/670	Survey time: 2013.06–2015.06 Mean age: 30.44 ± 3.56 Mean BMI: 24.16 ± 3.20 Menarche age: 13.23 ± 1.52 Primigravida:29.50% Diagnostic criteria: /	Low
Ginevra Mills et al (2020) [45]	Canada	2783/14882	Survey time: 2004–2014 Mean age:/ Mean BMI:/ Smoking: 4.6% Diagnostic criteria: /	High/acceptable
S. Weerakiet et al (2004) [43]	Thailand	8/47	Survey time: 1996.06–2002.05 Mean age: 31.6 ± 4 Mean BMI: 24.0 ± 3 Diagnostic criteria: 3-h 100-g OGTT at 24–28 weeks of gestation, ADA criteria	Low
Shiqiao Hu et al (2021) [26]	China	23/557	Survey time: 2016.01–2019.01 Mean age: 29.67 ± 3.57 Mean BMI: 22.63 ± 3.24 Diagnostic criteria: IADPSG criteria (2011)	High/acceptable
Hexia Xia et al (2017) [41]	China	31/94	Survey time: 2010.01–2014.12 Mean age: /Mean BMI: 23.96 Diagnostic criteria: IADPSG criteria (2011)	Low
Dayan Liu et al (2015) [58]	China	107/690	Survey time: 2012.01–2013.01 Mean age: 29.5 Mean BMI:24.7 Diagnostic criteria: 2-h 75-g OGTT	Low
Congcong Sun et al. (2019) [59]	China	54/114	Survey time: 2016.02–2018.08 Mean age: 29.1 Mean BMI: 23 Diagnostic criteria: 2-h 75-g OGTT at 24–28 weeks; 5.1–10.0–8.5 mmol/L	Low
Xiangzun Li et al (2017) [56]	China	12/35	Survey time: 2015.01–2017.01 Mean age: 31.5 ± 3.6 Mean BMI: 24.6 ± 2.6 Diagnostic criteria: 3-h 75-g OGTT at 24–28 weeks; 5.6–10.3–8.6–6.7 mmol/L	Low
Huizhuo Zhong et al. (2017) [57]	China	57/468	Survey time: 2010.01–2016.10 Mean age: / Mean BMI: /Diagnostic criteria: /	High/acceptable
Marlieke deWilde et al. (2015) [51]	The Netherlands	22/72	Survey time: Mean age: 30.5 Mean BMI: 27.4 Smoking: 9% Diagnostic criteria: ADA criteria (2003)	Low
R Helseth Vanky et al. (2013) [1]	Norway	67/273	Survey time:/ Mean age: 29.4 ± 4.4 Mean BMI: 29.0 ± 7.1 Smoking: 8.50% Diagnostic criteria: WHO criteria	High/acceptable
Guanghui Li et al (2018) [46]	China	75/248	Survey time: 2011–2013 Mean age: 30.43 Mean BMI: 24.60 Diagnostic criteria: FPG ≥ 5.1 mmol/L (92 mg/dL), 1‐hour plasma glucose ≥ 10.0 mmol/L (180 mg/dL), or 2‐hour plasma glucose ≥ 8.5 mmol/L (153 mg/dL)	High/acceptable
V. De Fre`ne et al. (2014) [27]	Belgium	119/200	Survey time: 2000.01–2009.12 Mean age: 28.7 Mean BMI: 25.8 Smoking: 20% Diagnostic criteria: ADA criteria (2014) Primigravida: 78.5% Irregular menstrual cycle: 94.5%	Low
Fatemeh Foroozanfard et al. (2020) [48]	Iran	3/43	Survey time: 2014.04–2016.04 Mean age: 24.58 Mean BMI: 24.20 Diagnostic criteria: /	High/acceptable
R. Bond et al. (2017) [53]	Canada	513/941	Survey time: 1990.01–2007.12 Mean age: /Mean BMI: /Diagnostic criteria:/ Primigravida:66.20%	Low
Mahnaz Ashrafi et al. (2014) [55]	Iran	104/234	Survey time: 2002–2003 Mean age: 29.6 ± 3.9 Mean BMI: 26.1 ± 3.4 Diagnostic criteria: ADA criteria (2005) Menarche age: 13.3 ± 1.5 Irregular mense: 84.60%	High/acceptable
Nadira Sultana Kakoly et al. (2017) [47]	Australia	63/448	Survey time: 1996–2012 Mean age: 36.6 ± 1.43 Mean BMI: 29.2 ± 7.86 SEI: 106 (16.0%, education < 12 y) Smoking: 9.52% Menarche age: 13.3 ± 1.5 Diagnostic criteria: /	Low
Michael Feichtinger et al. (2021) [49]	Austria	4/31	Survey time: 2015.06–2017.09 Mean age: 31.3 Mean BMI: /Diagnostic criteria: IADPSG criteria	Low
M.A. deWilde et al. (2014) [50]	The Netherlands	41/189	Survey time: 2008.04–2012.04 Mean age: 29 Mean BMI: 24 Smoking: 11.64% Diagnostic criteria: ADA criteria (2003)	High/acceptable
Roos N et al. (2011) [44]	Sweden	125/3787	Survey time: 1995–2007 Mean age: /Mean BMI: 24 Smoking: 9.57% Diagnostic criteria: as plasma glucose levels of 12.2 mmol/L or more after oral glucose tolerance test (75 g glucose orally administered and plasma glucose measured after two hours) or fasting blood glucose levels of 7.0 mmol/L or more	High/acceptable

*DM* Diabetes mellitus, *GDM* Gestational diabetes mellitus, *PCOS* Polycystic ovary syndrome, *BMI* Body mass index

*CI* Confidence interval, *WHO* World Health Organization, *ADA* American Diabetes Association, *IADPSG* criteria International Association of the Diabetes and Pregnancy Study Groups criteria

### Pooled incidence of gestational diabetes mellitus among women with polycystic ovary syndrome

**Fig. 2:**
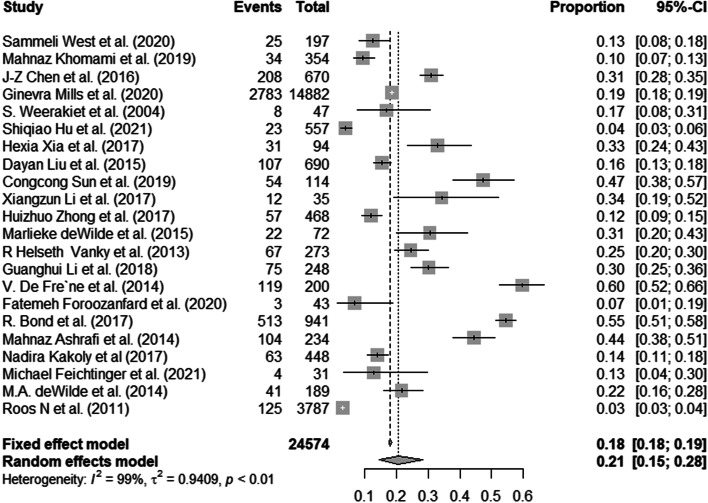
Forest plots of the included studies

A total of 22 studies reported incidence of GDM among women with PCOS. The forest plot was showed in Fig. 2 depicts the details. In the 22 studies, 24,574 women with polycystic ovary syndrome were included, of which 4478 were identified with GDM. The random effects model was used to calculate the pooled incidence (I^2^ = 98.80%, *P* < 0.001) in this review, and the pooled incidence of GDM among women with PCOS was 20.64%, with a 95% CI of 14.64% to 28.30%.

### The results of subgroup analysis

The results of subgroup analyses were showed in Table 2. Significant differences in the incidence of GDM between different age was found (*P* = 0.040). The results indicated that older PCOS patients showed higher incidence of GDM, younger participants (with a mean age ≤ 25) showed lowest incidence of GDM (6.98%). Although no significant difference in the incidence of GDM between different BMI group was found (*P* = 0.021), the results indicated that studies with higher percentage of overweight/obese patients showed higher incidence of GDM (*P* < 0.001). In addition, we found that studies with higher percentage of primigravida (> 30%) showed higher incidence of GDM (*P* < 0.001). Also, studies with higher percentage of smoking patients (> 10%) showed higher incidence of GDM (*P* = 0.044).

**Table 2:** The results of subgroup analysis

Subgroup	Studies	Pooled incidence % (95%CI)	I^2^ (%)	Test of Difference within Each Subgroup	
				Q	p
Mean age				8.08	0.040
≤ 25	1	6.98(2.27–19.51)	-		
26–30	7	26.34(13.66–44.69)	98.20		
31–35	8	20.78(14.56–28.75)	89.50		
> 35	1	14.06(11.14–17.60)	-		
Mean BMI				0.35	0.555
≤ 25	9	20.05 (14.44–39.05)	96.30		
> 25	7	24.74 (12.03–31.50)	96.80		
Percentage of overweight/obese patients				59.09	< 0.001
≤ 30%	2	18.74 (18.12–19.37)	0.00		
31%-40%	2	14.34 (11.52–17.72)	0.00		
41%-50%	2	28.30 (5.95–71.11)	98.50		
> 50%	1	40.37 (38.39–56.52)	-		
Percentage of primigravida				97.84	< 0.001
≤ 30%	1	31.04 (27.65–34.65)	-		
> 30%	2	55.39 (52.49–58.25)	0.00		
Percentage of smoking patients				4.05	0.044
≤ 10%	6	13.87 (7.61–23.96)	98.60		
> 10%	2	39.02 (16.66–67.18)	96.30		
WHO Area				5.33	0.255
European	7	19.06(9.36–34.95)	97.70		
Western Pacific	9	22.33(12.31–31.69)	97.30		
Americas	2	34.38(14.30–62.19)	99.70		
South-East Asia	2	14.34(11.52–17.72)	0.00		
Eastern Mediterranean	2	20.88(4.57–59.26)	87.60		
Income classification				0.08	0.783
High-income	11	19.74 (11.69–31.35)	99.10		
Upper-middle-income	11	21.65 (13.72–32.42)	97.00		
Sample size				4.26	0.038
0–300	13	27.40(19.91–26.41)	92.90		
≥ 301	9	14.02 (7.65–24.32)	99.40		
Quality score				3.03	0.081
Low	13	26.05 (18.07–36.01)	97.00		
High/acceptable	9	14.54 (20.04–25.00)	98.90		
Assessment tool				5.93	0.115
WHO criteria	2	15.71 (7.87–28.89)	91.70		
ADA criteria	5	33.80 (21.36–48.98)	93.10		
IADPSG criteria	3	12.37 (4.04–32.12)	93.10		
Others	6	18.90 (8.95–35.60)	98.20		

No significant differences in the incidence of GDM between different area was found (*P* = 0.255). Also, no significant differences in the incidence of GDM between different income group was found (*P* = 0.783). Additionally, significant difference in the incidence of GDM between included studies with different sample size was found, studies with bigger sample size (> 300) showed lower incidence of GDM (*P* = 0.038). For studies with different quality, the incidence of GDM in high-quality researches is lower than that of low-quality researches. However, the difference was not significant (*P* = 0.081). There was no significant difference between studies used different assessment tools (*P* = 0.115).

### The results of meta-regression analyses

The results of meta-regression analyses were reported in Table 3. Due to too many missing data on the percentage of overweight/obese patients, percentage of primigravida, percentage of smoking patients, this review was unable to include those variables in the regression model. The results of bivariate meta-regression indicated that higher incidence estimates reported in studies with smaller sample (β =  − 0.19, *p* = 0.041). Specifically, sample size accounted for 20.15% of the heterogeneity across the included studies. Also, studies which used ADA criteria as assessment tool showed higher incidence estimates (β =  − 0.21, *p* = 0.043). Specifically, sample size accounted for 22.11% of the heterogeneity across those included studies. Besides, area (β = -0.04, *p* = 0.676), quality score (β = -0.08, *p* = 0.422), mean BMI (β = 0.02, *p* = 0.513) and mean age (β = -0.06, *p* = 0.516) were not significant moderators.

**Table 3:** The results of meta-regression analysis

Group	β	95% CI		*p*	*R*^*2*^
		Lower	Upper		
Univariate analysis					
Area (Western Pacific region vs. others)	-0.04	-0.24	1.60	0.676	0.00%
Assessment tool **(**ADA criteria vs. others)	-0.21	-0.41	-0.01	0.043	22.11%
Sample (1–300 vs. ≥ 301)	-0.19	-0.37	-0.01	0.041	20.15%
Quality score (Low vs. High/acceptable)	-0.08	-0.29	0.12	0.422	0.00%
Mean age (continuous variable)	-0.06	-0.26	0.13	0.516	0.00%
Mean BMI (continuous variable)	0.02	-0.13	0.26	0.513	0.00%
Multivariate analysis					77.57%
Area (Western Pacific region vs. others)	-0.24	-0.43	-0.05	0.011	
Assessment tool **(**ADA criteria vs. others)	-0.16	-0.34	0.01	0.059	
Sample (1–300 vs. ≥ 301)	-0.39	-0.56	-0.23	< 0.001	
Quality score (Low vs. High/acceptable)	-0.12	-0.23	-0.01	0.039	
Mean age (continuous variable)	-0.08	-0.16	-0.01	0.028	
Mean BMI (continuous variable)	0.02	-0.01	0.06	0.220	

In the multivariate regression model, area (β = -0.24, *p* = 0.011), quality score of included studies (β = -0.12, *p* = 0.039), sample size (β = -0.39, *p* < 0.001) and mean age (β = -0.08, *p* = 0.028) were found as significant moderators for the heterogeneity (*P* < 0.05), accounted for 77.57% of the heterogeneity across studies.

### The results of sensitivity analysis and publication bias

When each study was excluded one-by-one, no significant changes were found among the recalculated combined incidences. The pooled incidence of GDM among PCOS patients ranged from 19.31% (95% CI: 13.78%-26.37%) to 22.44% (95% CI: 16.44%-26.86%), and the I^2^ statistic has ranged from 98.00% to 98.90%. The results in the current study indicate that no individual study significantly influenced the overall results. See Fig S1 for the details.

**Fig. 3:**
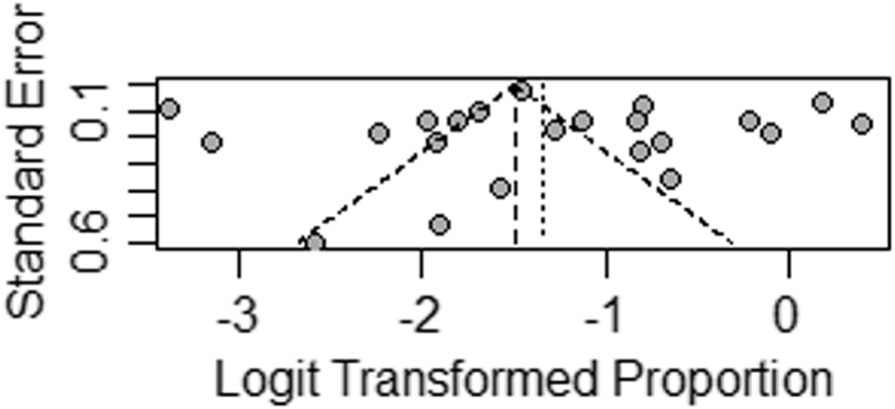
Funnel plot of publication bias

The funnel plot of publication bias is basically symmetric, but publication bias in this study cannot be ruled out based on it, so the Egger's test was performed. The results of the Egger's test indicated that publication bias was not exist in this study (t = 0.362, *p* = 0.721). See Fig. 3 for more details.

## Discussion

### Key findings

A total of 24,574 women with polycystic ovary syndrome were included in the 22 studies, of which 4478 were identified with gestational diabetes mellitus. The pooled incidence of GDM among women with PCOS was 20.64%, with a 95% CI of 14.64% to 28.30%. In the meta regression analyses, several variables including age, area, quality score and sample size were identified as significant factors of heterogeneity, accounted for 77.57% of the heterogeneity across studies.

#### Comparison with the literature

Previous study showed that the incidence of GDM among women with PCOS varies greatly across studies, ranged from 4.12% to 59.50%. The results in this study found that the pooled incidence of GDM among women with PCOS was 20.64%, provided a relatively accurate estimate, which may helpful for prevention of GDM and PCOS in the future. Based on the results of previous studies, the incidence of GDM in the general population was 4.40%-16.20% around the world [30, 60–62]. The pooled incidence of GDM among women with PCOS was 20.64%, which was much higher than the general population of pregnant women. This result suggests that more precautions should be taken against the occurrence of GDM in PCOS patients.

In the subgroup analysis, the incidence of gestational diabetes mellitus in older polycystic ovary syndrome patients was significantly higher than the younger patients, which was consistent with other researches [63]. The risk of GDM increases linearly with age, which has been reported in the previous literature. The mechanism of the association between maternal age and GDM is not yet clear. High level of insulin resistance, high levels of circulating adipokines and inflammatory markers as well as oxidative stress may partly explain this phenomenon [64, 65]. In addition, we also found that the incidence of GDM among PCOS patients declined a lot after 35 years of age in the subgroup analysis, and after controlling for other factors, the results of the regression model showed that older age was associated with lower incidence of GDM. This result indicated that the occurrence of PCOS among PCOS patients cannot be simply attributed to maternal age. Thus, the association between age and the incidence of GDM in the current review needs further exploration.

Several studies have showed associations between overweight/obesity and the development of GDM among pregnant women [7, 30, 61, 66]. In the current study, association between overweight/obesity and the incidence of GDM was significant in the univariate analysis. When controlled the influence of other factors in the multivariate analysis, the difference is no longer significant. In addition, our study showed that higher percentage of primigravida was associated with high incidence of GDM. The possible reason is that primigravida have no experience for gestation, it is more difficult for them to stay healthy during pregnancy. Due to too many missing data, this variable could not be included in the meta-regression model. Therefore, this observation needs further clarification. Smoking during pregnancy is said to be associated with an increased risk of GDM [67]. A possible explanation of the association is the effect of smoking on increased oxidative stress, inflammation, hyperglycemia, and insulin resistance, but the exact mechanism of action is yet to be determined [68]. In the current study, smoking during pregnancy is associated with an increased risk for GDM among PCOS patients too. Due to too many missing data, smoking could not be included in the meta regression model, the current results may be required further exploration.

There was significant difference between different regions in the meta-regression model, we have no clear reason for such a discrepancy, but we speculate that it may due to differences in the ethnic background [7]. Considering that none of the included studies were conducted in low-income countries, which may be related to the number of studies, we believe that more studies are needed in low-income countries to understand the full picture of GDM in PCOS patients. Furthermore, we found that the pooled incidence of gestational diabetes mellitus among polycystic ovary syndrome patients identified by different assessment tools was not significant. To date, the criteria for gestational diabetes mellitus (GDM) screening and diagnosis are controversial around the world, different countries use different diagnostic criteria to determine the incidence of GDM [69]. Inconsistencies in the GDM screening strategy between different guidelines have led to challenges in estimating the incidence, future study is needed to explore international standards for the ascertainment of GDM. It is reported that studies with poor methodological quality and small sample size usually yielded more extreme results [70], the current review observed similar results, studies with small sample size and poorer methodological quality reported higher incidence of GDM.

### Implications for the future

During the process of screening data, we found that there were relatively few data on incidence of GDM among PCOS patients. Of the 22 included studies, 13 (59.09%) were rated as low quality and 59.09% of the included studies with a sample size ≤ 300. Therefore, it is necessary to conduct a large multi-center prospective research in the future, use a validated measure of GDM in randomly selected PCOS patients. This kind of study should measure possible confounding factors in the future, which will provide a more accurate incidence of GDM among PCOS patients. Currently, the results of some studies reported that dietary or combined lifestyle measures have not indicated too much improvements in the risk of developing GDM. Besides, those studies involving physical activity programs have yielded conflicting results [71]. Given the great potential for reducing the disease burden of PCOS patients, future research should continue to identify interventions that can be easily implemented in patients with PCOS, especially during their preconception period. Additionally, due to lack of data in many subgroups, we were unable to perform meta regression analysis for some possible confounders, such as socioeconomic status, family history of GDM, physical activity, drinking and diet habit [6, 54]. Accordingly, there might be considerable uncertainty regarding the pooled incidence of GDM among PCOS patients. Future researchers should explore more potential risk factors for GDM among PCOS patients, especially genetic background as well as health-related behavior or other concomitant chronic diseases.

### Limitations

Although a protocol was conducted before the review was started, the protocol was not published or registered, which is a limitation of this study. Subgroup analyses and meta regression were conducted to control many factors for the pooled incidence of GDM among PCOS patients, however, heterogeneity was found in the current study. Previous studies have demonstrated that heterogeneity is very difficult to avoid in meta-analysis of observational studies [72]. Besides, papers not written in English or Chinese were excluded, which is also a limitation of this review. Additionally, although this review included studies across 11 countries / areas, most of the eligible articles were conducted in high income countries/areas, no study was conducted in low-income country/area. Considering the inconsistency of the economic status and health care environment worldwide, more incidence studies in low-income countries/areas are needed to understand the full picture of GDM among PCOS patients. Also, we noticed that the included studies covering a vast range of clinical and diagnostic criteria and practice changes [63]. It is possible that the pooled incidence of GDM among PCOS patients was influenced by the changes of threshold value to identify GDM. Thus, we think ongoing surveillance is essential.

## Conclusion

A total of 24,574 women with polycystic ovary syndrome were included in the 22 studies, of which 4478 were identified with gestational diabetes mellitus. The pooled incidence of gestational diabetes mellitus among women with polycystic ovary syndrome was 20.64%, with a 95% CI of 14.64% to 28.30%. In the meta regression analyses, several variables including age, area, quality score and sample size were found as significant sources of heterogeneity, accounted for 77.57% of the heterogeneity across studies. More study is needed to explore possible risk factors for GDM and identify effective strategies for preventing GDM among PCOS patients.

## Supplementary Information

**Additional file 1.** 

## Data Availability

Data supporting the findings of this review were presented in Table 1 and Supplementary Data.

## Abbreviations

DM: Diabetes mellitus
GDM: Gestational diabetes mellitus
PCOS: Polycystic ovary syndrome
BMI: Body mass index
CI: Confidence interval
WHO: World Health Organization
ADA: American Diabetes Association
IADPSG criteria: International Association of the Diabetes and Pregnancy Study Groups criteria
