# Supplementary material for: The incidence of gestational diabetes mellitus among women with polycystic ovary syndrome: a meta-analysis of longitudinal studies
Source: BMC Pregnancy Childbirth. 2022 Apr 29;22:370. doi: 10.1186/s12884-022-04690-3 (PMC9055740; doi:10.1186/s12884-022-04690-3)
Supplement: Supplementary file 1 — Additional file 1. [file 12884_2022_4690_MOESM1_ESM.pdf]

Supplementary data

Search strategy

Table S1 Search strategy for PubMed:

**a)**	**Polycystic Ovary Syndrome**
1.	Polycystic Ovary Syndrome [Title/Abstract]
2.	PCOS [Title/Abstract]
3.	polycystic ovarian syndrome [Title/Abstract]
4.	poly cystic ovarian syndrome [Title/Abstract]
5.	polycystic ovary disease [Title/Abstract]
6.	Ovarian Cysts [Title/Abstract]
7.	Stein Leventhal Syndrome [Title/Abstract]
8	1 OR 2 OR 3 OR 4 OR 5 OR 6 OR 7
**b)**	**Gestational diabetes mellitus**
**9.**	gestational diabetes mellitus [Title/Abstract]
10.	gestational diabetes [Title/Abstract]
11.	insulin dependent diabetes [Title/Abstract]
12.	Getational* [Title/Abstract]
13.	non-insulin dependent diabetes [Title/Abstract]
14.	pregnancy-induced diabetes [Title/Abstract]
15	GDM [Title/Abstract]
16.	10 OR 11 OR 12 OR 13 OR 14 OR 15
**c)**	**Longitudinal study**
17.	Longitudinal study [Title/Abstract]
18.	Longitudinal Survey [Title/Abstract]
19.	Follow up study [Title/Abstract]
20	Cohort study [Title/Abstract]
**21.**	Epidemiologic Studies [Title/Abstract]
**22.**	observational study [Title/Abstract]
23	17 OR 18 OR 19 OR 20 OR 21 OR 22
24.	a) AND b) AND c)

Table S2 The items of quality assessment

	Item
	(i) Can we be confident in the assessment of exposure (ie, the predictor variables)?
	(ii) Were the exposed and non-exposed cohorts selected from the same population?
	(iii) Can we be confident that the outcome of interest was not present at the start of the study?
	(iv) Did the statistical analysis adjust for the confounding variables?
	(v) Can we be confident in the assessment of the presence or absence of confounding factors?
	(vi) Can we be confident in the assessment of the outcome?
	(vii) Was the follow-up of the cohorts adequate?

Table S3 The results of quality assessment

study	Confidence in exposure (predictor) assessment	Exposed and unexposed from the same population	Confidence in exclusion of prevalent cases	Comprehensive adjustments	Confidence in confounders assessment	Confidence in outcome assessment	Adequate follow-up	High/acceptable quality
Sammeli West et al. (2020)	+	++	-	+	+	-	+	No
Mahnaz Khomami et al. (2019)	++	++	-	+	+	++	+	No

J-Z Chen et al. (2016)	++	++	-	++	++	++	++	No
Ginevra Mills et al. (2020)	++	++	+	++	+	+	++	Yes
S. Weerakiet et al. (2004)	++	++	-	+	+	++	++	No
Shiqiao Hu et al. (2021)	++	++	++	+	+	++	++	Yes
Hexia Xia et al. (2017)	++	++	-	+	++	++	+	No
Dayan Liu et al. (2015)	+	++	++	-	-	++	+	No
Congcong Sun et al. (2019)	++	++	++	+	-	++	-	No
Xiangzun Li et al. (2017)	++	++	++	+	-	++	+	No
Huizhuo Zhong et al. (2017)	++	++	++	+	+	++	+	Yes
Marlieke deWilde et al. (2015)	++	++	-	-	+	+	+	No
R Helseth, E Vanky et al. (2013)	++	++	++	+	++	++	+	Yes
Guanghui Li et al. (2018)	+	++	++	+	++	++	+	Yes
V. De Fre`ne et al.	++	++	-	+	++	++	+	No

(2014)								
Fatemeh Foroozanfard et al. (2020)	++	++	+	+	-	++	-	Yes
R. Bond et al. (2017)	+	++	++	+	++	++	-	No
Mahnaz Ashrafi et al. (2014)	++	++	++	+	+	++	+	Yes
Nadira Sultana Kakoly et al (2017)	+	++	-	+	++	+	++	No
Michael Feichtinger et al. (2021)	++	++	++	+	-	++	+	No
M.A. deWilde et al. (2014)	++	++	+	++	++	++	++	Yes
Roos N et al. (2011)	++	++	+	++	++	++	++	Yes

Note: Symbols ++ = definitely yes; + = probably/mostly yes; - = probably/mostly no; - - = definitely no

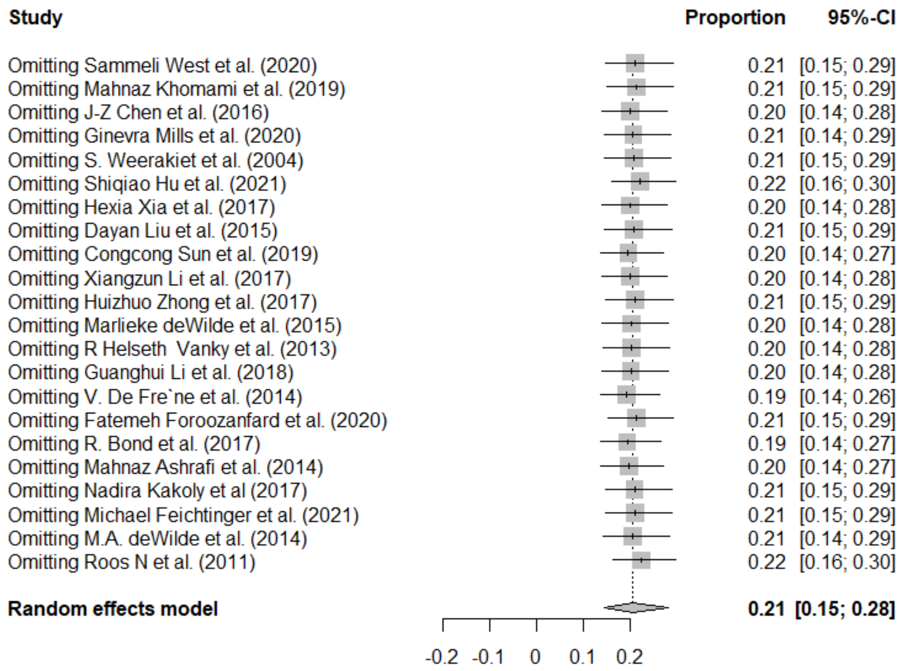

Fig. S1 Forest plots for the sensitivity analysis

Table S4 The categories of subgroups

Variable	Reason for classification	Reference
Age	Age was significantly associated with the occurrence of GDM, with large changes every 5 years. Based on this result, we grouped this variable into four groups (≤25 vs. 26-30 vs. 31-35 vs. >35)	[Yueyi Li] Maternal age and the risk of gestational diabetes mellitus: A systematic review and meta-analysis of over 120 million participants
Mean BMI	There was a significant difference in the incidence of GDM between participants with a BMI ≥ 25 and a BMI less than 25. So we grouped this variable according to previous findings.	[Kai Wei Lee] Prevalence and risk factors of gestational diabetes mellitus in Asia: a systematic review and meta-analysis
Percentage of	Previous study showed that obesity has a	[Panagiotis Anagnostis] Risk of type 2

overweight/obese patients	large effect on the occurrence of GDM, so we grouped this variable according to experience and the distribution of our data.	diabetes mellitus in polycystic ovary syndrome is associated with obesity: a meta-analysis of observational studies
Percentage of primigravida	Previous study indicated the importance of this variable. We therefore decided to explore the effect of this factor on incidence.<br>Only 3 articles reported relevant data (the percentage of primigravida is 29.5%, 78.5% and 66.2%, respectively), we grouped this variable according to the distribution of the data.	[Kai Wei Lee] Prevalence and risk factors of gestational diabetes mellitus in Asia: a systematic review and meta-analysis
Percentage of smoking patients	Previous study indicated that the estimated prevalence of smoking during pregnancy was more than 10% in 29 (17%) of 174 countries. Based on this data, we grouped this variable in to two group (≤10% vs. >10%)	[Shannon Lange] National, regional, and global prevalence of smoking during pregnancy in the general population: a systematic review and meta-analysis
WHO Area	The occurrence of GDM varies greatly in different regions, so we grouped regions according to WHO classification as a subgroup	[Yeyi Zhu] Prevalence of Gestational Diabetes and Risk of Progression to Type 2 Diabetes: a Global Perspective
Income classification	Income classification of each country based on the World Bank classification (high-income vs. upper-middle-income vs. lower-middle-income vs. low-income countries)	[Yeyi Zhu] Prevalence of Gestational Diabetes and Risk of Progression to Type 2 Diabetes: a Global Perspective
Sample size	As one of the important variables in assessing risk of bias, we decided to explore the effect of sample size on incidence.<br>Of 59.09% (13/22) of the included studies with a sample size ≤ 300, we grouped this variable according to the distribution of the data.	
Quality score	Previous studies highlighted the impact of assessment tools on outcome, so we grouped quality score based on Virtanen et al.'s experience.	[Marianna Virtanen] Long working hours and depressive symptoms: systematic review and meta-analysis of published studies and unpublished individual participant data
Assessment tool	Previous studies highlighted the impact of assessment tools on incidence, so we grouped assessment tools based on prior experience.	[Kai Wei Lee] Prevalence and risk factors of gestational diabetes mellitus in Asia: a systematic review and meta-analysis;<br>[Maryam Saeedi] Increasing

		prevalence of gestational diabetes mellitus when implementing the IADPSG criteria: A systematic review and meta-analysis

Table S5 PRISMA checklist

Section / topic	#	Checklist item	Reported on page #
**TITLE**			
Title	1	The incidence of gestational diabetes mellitus among women with polycystic ovary syndrome: a meta-analysis of longitudinal studies	Title
**ABSTRACT**			
Structured summary	2	Background: Previous studies have shown that PCOS is a predictor of GDM, but we do not know exactly how many PCOS patients may develop GDM. Currently, the incidence of GDM among women with PCOS varies greatly across studies, ranged from 4.12% to 59.50%. In addition, many factors have been reported to be associated with the incidence of GDM among women with PCOS, but the results are not consistent in different studies. The possible causes of the inconsistencies in the current estimates were unclear. This review aimed at examining the pooled incidence of GDM among women with PCOS, summarizing possible vulnerability factors of GDM among women with PCOS, try to provide a reference for prevention of GDM and PCOS in the future.<br><br>Methods: Systematic searches of databases were conducted for literature published until 31 May 2021. Statistical analyses were performed using R software, the pooled incidence was combined using random effects model. Cochrane's "Tool to Assess Risk of Bias in Cohort Studies" was used for quality assessment.<br><br>Results: Twenty-two longitudinal studies were included. A total of 24574 women with polycystic ovary syndrome were identified in the 22 articles, of which 4478 were reported with gestational diabetes mellitus. The pooled incidence of gestational diabetes mellitus among women with polycystic ovary syndrome was 20.64%, with a 95% CI of 14.64% to 28.30%. In the meta-regression model, several variables including age, area, quality score and sample size were found as significant sources of heterogeneity, accounted for 77.57% of the heterogeneity across studies.	Abstract

		Conclusions: Evidence in this review suggests that gestational diabetes mellitus were common among women with polycystic ovary syndrome. Further research is needed to identify effective strategies for preventing gestational diabetes mellitus among women with polycystic ovary syndrome.	
**INTRODUCTION**			
Rationale	3	Previous studies have shown that PCOS is a predictor of GDM, but we do not know exactly how many PCOS patients may develop GDM. Currently, the incidence of GDM among women with PCOS varies greatly across studies, ranged from 4.12% to 59.50%. In addition, many factors have been reported to be associated with the incidence of GDM among women with PCOS, but the results are not consistent in different studies. The possible causes of the inconsistencies in the current estimates were unclear. This review aimed at examining the pooled incidence of GDM among women with PCOS, summarizing possible vulnerability factors of GDM among women with PCOS, try to provide a reference for prevention of GDM and PCOS in the future.	Introduction
Objectives	4	This review aimed at examining the pooled incidence of GDM among women with PCOS, summarizing possible vulnerability factors of GDM among women with PCOS, try to provide a reference for prevention of GDM and PCOS in the future.	Introduction
**METHODS**			
Protocol and registration	5	This review was reported in accordance with the PRISMA guideline and MOOSE guidelines.	Methods
Eligibility criteria	6	Studies were included if they meet the following criteria: (1) the study was longitudinal observational study; (2) the participants were women with polycystic ovary syndrome; (3) information about incidence of gestational diabetes mellitus among women with polycystic ovary syndrome was provided; (4) the full article was written in English or Chinese. Studies were excluded if (1) the report was a meta-analysis, review, conference abstract, comments, or protocol.	Methods
Information sources	7	PubMed, the Cochrane Library, EMBASE, Web of Science, MEDLINE, Chinese National Knowledge Infrastructure (CNKI) were independently searched for published articles by two reviewers, with no restrictions on date or language of publication up until 31 May 2021. The following search terms were used: ‘Polycystic Ovary Syndrome’ (including ‘Polycystic Ovary Syndrome’, ‘PCOS’, ‘polycystic ovarian syndrome’, ‘polycystic ovary disease’, ‘Ovarian Cysts’, ‘Stein Leventhal Syndrome’, and ‘poly cystic ovarian syndrome’.); “gestational diabetes mellitus” (including ‘gestational diabetes mellitus’, ‘gestational diabetes’, ‘GDM’, ‘gestational’,	Methods

		'insulin dependent diabetes', 'non-insulin dependent diabetes' and 'pregnancy-induced diabetes'); Longitudinal study (including 'longitudinal study', 'longitudinal Survey', 'follow up study', 'cohort study', 'epidemiologic Studies' and 'observational study'). See supplementary data for a full search strategy.	
Search	8	a) Polycystic Ovary Syndrome<br>1. Polycystic Ovary Syndrome [Title/Abstract]<br>2. PCOS [Title/Abstract]<br>3. polycystic ovarian syndrome [Title/Abstract]<br>4. poly cystic ovarian syndrome [Title/Abstract]<br>5. polycystic ovary disease [Title/Abstract]<br>6. Ovarian Cysts [Title/Abstract]<br>7. Stein Leventhal Syndrome [Title/Abstract]<br>8 1 OR 2 OR 3 OR 4 OR 5 OR 6 OR 7<br>b) Gestational diabetes mellitus<br>9. gestational diabetes mellitus [Title/Abstract]<br>10. gestational diabetes [Title/Abstract]<br>11. insulin dependent diabetes [Title/Abstract]<br>12. Getational* [Title/Abstract]<br>13. non-insulin dependent diabetes [Title/Abstract]<br>14. pregnancy-induced diabetes [Title/Abstract]<br>15 GDM [Title/Abstract]<br>16. 10 OR 11 OR 12 OR 13 OR 14 OR 15<br>c) Longitudinal study<br>17. Longitudinal study [Title/Abstract]<br>18. Longitudinal Survey [Title/Abstract]<br>19. Follow up study [Title/Abstract]<br>20 Cohort study [Title/Abstract]<br>21. Epidemiologic Studies [Title/Abstract]<br>22. observational study [Title/Abstract]<br>23 17 OR 18 OR 19 OR 20 OR 21 OR 22<br>24. a) AND b) AND c)	Supplementary data
Study selection	9	Data extraction was conducted independently in pairs by trained researchers who used standardized data extraction forms. Two reviewers (QZY and DQ) checked the titles, abstracts and full-texts of the initial search results independently. Data were extracted on first author, country/area, publication year, sample size, mean age, mean BMI, percentage of overweight/obese patients, percentage of primigravida, percentage of smoking participants, mean age of participants, instruments used to identify GDM, incidence of GDM, and quality score of the included studies. Any discrepancies that emerged in these procedures were discussed and resolved by involving a third reviewer (XL).	Methods

Data collection process	10	Data extraction was conducted independently in pairs by trained researchers who used standardized data extraction forms. Two reviewers (QZY and DQ) checked the titles, abstracts and full-texts of the initial search results independently. Any discrepancies that emerged in these procedures were discussed and resolved by involving a third reviewer (XL).	Methods
Data items	11	Data were extracted on first author, country/area, publication year, sample size, mean age, mean BMI, percentage of overweight/obese patients, percentage of primigravida, percentage of smoking participants, instruments used to identify GDM, incidence of GDM, and quality score of the included studies.	Methods
Risk of bias in individual studies	12	Two independent reviewers (RZL and YXH) used the established guidelines, Cochrane's "Tool to Assess Risk of Bias in Cohort Studies", to evaluate the methodological quality of the included studies, which has been widely used to evaluate observational studies	Methods
Summary measures	13	Incidence of GDM	Methods
Synthesis of results	14	When data were available for three or more papers, incidence of gestational diabetes mellitus was combined (32). When there were 4 or more studies available, quantitative subgroup analysis was conducted (33). All the statistical analyses in this study were performed using the "meta" (4.13-0) and "metafor" package (2.4-0) of R version 4.0.0. Heterogeneity between the included studies was evaluated by Cochran's Q test and quantified by the I2 statistic. When the results of I2 greater than 50%, means moderate heterogeneity (33). As the authors expected considerable heterogeneity, pooled incidence of gestational diabetes mellitus was calculated with the random effects model (34). The pooled incidence of gestational diabetes mellitus among women with polycystic ovary syndrome was combined using Logit transformation method by a random effects model in the current study. In order to compare the incidence of gestational diabetes mellitus from different studies, subgroup analysis was conducted. Previous research indicated that subgroup analyses should be interpreted with caution, we planned a priori to limit our subgroup analyses to a limited number of baseline characteristics including area, mean age, mean BMI, percentage of overweight/obese patients, percentage of primigravida, percentage of smoking patients, sample size, and quality score (34). The difference between those subgroups was examined using the Cochran's Q chi-square tests. Mixed-model meta-regression analyses were performed by using Freeman-Tukey double arcsine method to explore potential moderators on the heterogeneity. Publication bias was investigated by funnel plot and Egger's test. To evaluate the consistency of the results, sensitivity analysis was performed. In this study, sensitivity analyses	Methods

		were planned a priori for the primary analyses set by excluding studies one by one. All the statistical tests were 2-sided, with a significance threshold of $P < 0.05$.	

Section/topic	#	Checklist item	Reported on page #
Risk of bias across studies	15	Publication bias was investigated by funnel plot and Egger's test. To evaluate the consistency of the results, sensitivity analysis was performed by removing each study individually. All the statistical tests were 2-sided, with a significance threshold of $P < 0.05$.	Methods
Additional analyses	16	In order to compare the incidence from different studies (such as age, area, diagnostic method, BMI etc.), we conducted subgroup meta-analysis. The difference between subgroups was examined using the Cochran's Q chi-square tests. Mixed-model meta-regression analyses were performed by using Freeman-Tukey double arcsine method to explore potential moderators on the heterogeneity.	Methods
**RESULTS**			
Study selection	17	As reported in Fig. 1, a total of 616 references were identified. Among those references, 95 duplicates were removed. By screening titles and abstracts, 445 irrelevant articles were excluded. A total of 76 potentially relevant full-text articles were independently assessed based on the selection criteria. Further, 54 studies were excluded because of the following reasons: duplicate articles or results (n = 8); review or conference abstract (n = 4); did not provide data on incidence of gestational diabetes mellitus among women with polycystic ovary syndrome (n = 32); not observational study (n = 7); unable to locate full text (n = 3). Finally, 22 eligible studies were included in this review. See Fig. 1 for the details.	Results
Study characteristics	18	Table 1 presents the main characteristics of the 22 included studies (24, 25, 35-54). Among them, 18 were in English and 4 were in Chinese. Most of the included studies were from Asia and European, such as China, Finland and Canada. See Table 1 for the details. From the 22 studies, 9 (40.91%) studies were rated as high or acceptable quality and 13 (59.09%) were rated as low quality. Addition, 59.09% (13/22) of the included studies with a sample size $\leq$ 300. Details of the methodological quality assessments of all 22 studies are showed in Table S2 and Table S3.	Results
Risk of bias within studies	19	From the 22 studies, 9 (40.91%) studies were rated as high or acceptable quality and 13 (59.09%) were rated as low quality. Details of the methodological quality assessments of all 22 studies are showed in Additional File 2.	Results
Results of	20	There were 22 studies reported incidence of gestational diabetes	Results

individual studies		mellitus among women with polycystic ovary syndrome. The forest plot in Fig. 2 depicts the details. A total of 24574 women with polycystic ovary syndrome were identified in the 22 articles, of which 4478 were reported with gestational diabetes mellitus.	
Synthesis of results	21	There were 22 studies reported incidence of gestational diabetes mellitus among women with polycystic ovary syndrome. The forest plot in Fig. 2 depicts the details. A total of 24574 women with polycystic ovary syndrome were identified in the 22 articles, of which 4478 were reported with gestational diabetes mellitus. The random effects model was used to determine the pooled incidence ($Q= 1997.85$, $I2 = 98.80\%$, $P < 0.001$), the pooled incidence of gestational diabetes mellitus among women with polycystic ovary syndrome was 20.64%, with a 95% CI of 14.64% to 28.30%.	Results
Risk of bias across studies	22	Funnel plot of publication bias is presented in Fig. 3. The funnel plot of publication bias is basically symmetric, but publication bias cannot be ruled out, so Egger's test was conducted. The results of the Egger's test showed that publication bias was not found in this study ($t = 0.362$, $p = 0.721$).<br>When each study was excluded one-by-one, the recalculated combined results did not change significantly. The pooled incidence of GDM among PCOS patients ranged from 19.31% (95% CI: 13.78%-26.37%) to 22.44% (95% CI: 16.44%-26.86%), and the I2 statistic has ranged from 98.00% to 98.90%. The results in the current study indicate that no individual study significantly influenced the overall results. See Fig S1 for the details of sensitivity analysis.	Results
Additional analysis	23	The details of subgroup analyses are presented in Table 2. Significant differences in the incidence of gestational diabetes mellitus between different age was found ($Q=8.08$, $P = 0.040$). The results indicated that older polycystic ovary syndrome patients showed higher incidence of gestational diabetes mellitus, younger participants (with a mean age ≤25) showed lowest incidence of gestational diabetes mellitus (6.98%). Although no significant difference in the incidence of gestational diabetes mellitus between different BMI group was observed (20.05% vs. 24.74%; $Q= 5.31$, $P = 0.021$), the results indicated that studies with higher percentage of overweight/obese patients showed higher incidence of gestational diabetes mellitus (18.74% vs. 14.34% vs. 28.30% vs. 40.37%; $Q= 59.09$, $P < 0.001$). In addition, we found that studies with higher percentage of primigravida (> 30%) showed higher incidence of gestational diabetes mellitus (31.04% vs. 55.39%; $Q= 97.84$, $P < 0.001$). Also, studies with higher percentage of smoking patients (>10%) showed higher incidence of gestational diabetes mellitus (13.87% vs. 39.02%; $Q= 4.05$, $P = 0.044$)<br>The pooled incidence of gestational diabetes mellitus among polycystic ovary syndrome patients in the European region, the Western Pacific	

	24	region, the America region, the South-East Asia region and the Eastern Mediterranean region was 19.06%, 22.33%, 34.38%, 14.34% and 20.88%, respectively. No significant differences in the incidence of gestational diabetes mellitus between different region was found (Q= 5.33, P = 0.255). Furthermore, the pooled incidence of gestational diabetes mellitus among patients in the high-income region and the upper-middle-income region was 19.74% and 21.65%, respectively. No significant differences in the incidence of gestational diabetes mellitus between different income classification group was found (Q= 0.08, P = 0.783). Additionally, significant difference in the incidence of gestational diabetes mellitus between studies with different sample size was observed, articles with higher sample size (>300) showed lower incidence of gestational diabetes mellitus (27.40% vs. 14.02%; Q= 4.26, P = 0.038). For studies with different quality, the incidence of gestational diabetes mellitus in high-quality researches is lower than that of low-quality researches. However, the difference was not significant (26.05% vs. 14.54%; Q= 3.03, P = 0.081).<br>Table 3 showed the results of meta-regression analyses. Due to too many missing data on the percentage of overweight/obese patients, percentage of primigravida, percentage of smoking patients, we were unable to include those variables in the meta-regression model. Bivariate meta-regression suggested that higher incidence estimates reported in studies with small sample ($\beta = -0.19$, $p = 0.041$). Specifically, sample size accounted for 20.15% of the heterogeneity across studies. Also, higher incidence estimates reported in studies which used ADA criteria as assessment tool ($\beta = -0.21$, $p = 0.043$). Specifically, sample size accounted for 22.11% of the heterogeneity across studies. Besides, area ($\beta = -0.04$, $p = 0.676$), quality score (β = -0.08, $p = 0.422$), mean BMI ($\beta = 0.02$, $p = 0.513$) and mean age (β = -0.06, $p = 0.516$) and were not significant moderators.<br>Of the multivariate model, area (β = -0.24, $p = 0.011$), quality score (β = -0.12, $p = 0.039$), sample size (β = -0.39, $p < 0.001$) and mean age (β = -0.08, $p = 0.028$) were found as significant moderators for heterogeneity ($P < 0.05$), accounted for 77.57% of the heterogeneity across studies.	
**DISCUSSION**			
Summary of evidence	24	A total of 24574 women with polycystic ovary syndrome were identified in the 22 articles, of which 4478 were reported with gestational diabetes mellitus. The pooled incidence of gestational diabetes mellitus among women with polycystic ovary syndrome was 20.64%, with a 95% CI of 14.64% to 28.30%. In the meta regression analyses, several variables including age, area, quality score and sample size were found as significant sources of heterogeneity, accounted for 77.57% of the heterogeneity across studies.	Discussion

Limitations	25	Firstly, we excluded papers were not written in English or Chinese. Besides, although subgroup analyses were conducted to control many moderating factors for the pooled incidence of GDM among PCOS patients, heterogeneity remained in this review. It is reported that heterogeneity is difficult to avoid in meta-analysis of epidemiological surveys (68), which suggesting the need for caution when drawing inferences about estimates of GDM among PCOS patients. Additionally, although this review included relevant studies across 11 countries, most of the eligible studies were from high income countries, no study was conducted in low-income country. Considering the inconsistency of the health care environment and economic status worldwide, more incidence studies in low-income countries are needed to understand the panorama of GDM among PCOS patients. Also, we noticed that the included studies covering a vast range of clinical and diagnostic criteria and practice changes (58). It is possible that the pooled incidence of GDM among PCOS patients was influenced by the changes of threshold value to identify GDM. Thus, we think ongoing surveillance is essential.	Discussion
Conclusions	26	A total of 24574 women with polycystic ovary syndrome were identified in the 22 articles, of which 4478 were reported with gestational diabetes mellitus. The pooled incidence of gestational diabetes mellitus among women with polycystic ovary syndrome was 20.64%, with a 95% CI of 14.64% to 28.30%. In the meta regression analyses, several variables including age, area, quality score and sample size were found as significant sources of heterogeneity, accounted for 77.57% of the heterogeneity across studies. Further research is needed to explore more possible risk factors for GDM and identify effective strategies for preventing GDM among PCOS patients.	Discussion
**FUNDING**			
Funding	27	This research was supported by the Health Commission of Hunan Province (Grant NO: B2017167) and Hunan Pharmaceutical Association (Grant NO: Hn2017007).	Funding

From: Moher D, Liberati A, Tetzlaff J, Altman DG, The PRISMA Group (2009). Preferred Reporting Items for Systematic Reviews and Meta-Analyses: The PRISMA Statement. PLoS Med 6(6): e1000097. doi:10.1371/journal.pmed1000097

For more information, visit: **www.prisma-statement.org**.

Table S6 MOOSE Checklist

Criteria		Brief description of how the criteria were handled in the meta-analysis
**Reporting of background should include**		
√	Problem definition	Previous studies have shown that PCOS is a predictor of GDM, but we do not know exactly how many PCOS patients may develop GDM. Currently, the incidence of GDM among women with PCOS varies greatly across studies, ranged from 4.12% to 59.50%. In addition, many factors have been reported to be associated with the incidence of GDM among women with PCOS, but the results are not consistent in different studies. The possible causes of the inconsistencies in the current estimates were unclear.
√	Hypothesis statement	GDM are very common among PCOS patients, relevant study characteristics, such as area, outcome measures, age, BMI have an impact on the outcome.
√	Description of study outcomes	Incidence of GDM
√	Type of exposure or intervention used	GDM
√	Type of study designs used	We included longitudinal studies
√	Study population	PCOS patients
**Reporting of search strategy should include**		
√	Qualifications of searchers	The credentials of the two investigators DQ and QZY are indicated in the author list.
√	Search strategy, including time period included in the synthesis and keywords	The following search terms were used: 'Polycystic Ovary Syndrome' (including 'Polycystic Ovary Syndrome', 'PCOS', 'polycystic ovarian syndrome', 'polycystic ovary disease', 'Ovarian Cysts', 'Stein Leventhal Syndrome', and 'poly cystic ovarian syndrome'.); " gestational diabetes mellitus" (including 'gestational diabetes mellitus', 'gestational diabetes', 'GDM', 'gestational', 'insulin dependent diabetes', 'non-insulin dependent diabetes' and 'pregnancy-induced diabetes'); Longitudinal study (including 'longitudinal study', 'longitudinal Survey', 'follow up study', 'cohort study', 'epidemiologic Studies' and 'observational study'). See supplementary data for a full search strategy.
√	Databases and registries searched	PubMed, the Cochrane Library, EMBASE, Web of Science, MEDLINE, Chinese National Knowledge Infrastructure (CNKI) were independently searched for

		published articles by two reviewers, with no restrictions on date or language of publication up until 31 May 2021.
√	Search software used, name and version, including special features	We did not employ a search software. EndNote was used to merge retrieved citations and eliminate duplications
√	Use of hand searching	We hand-searched bibliographies of retrieved papers for additional references
√	List of citations located and those excluded, including justifications	Details of the literature search process are outlined in the flow chart and supplementary data. The citation list is available upon request
√	Method of addressing articles published in languages other than English	We excluded studies not in English and Chinese
√	Method of handling abstracts and unpublished studies	We planned to contacted authors for unpublished studies during the screening process when necessary, no such abstracts and unpublished studies appears in articles that meet the inclusion criteria at last.
√	Description of any contact with authors	Not applicable (All articles that meet the inclusion criteria have complete data for pooled prevalence)
**Reporting of methods should include**		
√	Description of relevance or appropriateness of studies assembled for assessing the hypothesis to be tested	Detailed inclusion and exclusion criteria were described in the methods section.
√	Rationale for the selection and coding of data	Two reviewers (QZY and DQ) checked the titles, abstracts and full-texts of the initial search results independently. Data were extracted on first author, country/area, publication year, sample size, mean age, mean BMI, percentage of overweight/obese patients, percentage of primigravida, percentage of smoking participants, instruments used to identify GDM, incidence of GDM, and quality score of the included studies. Any discrepancies that emerged in these procedures were discussed and resolved by involving a third reviewer (XL).
√	Assessment of confounding	In order to compare the incidence of gestational diabetes mellitus from different studies, subgroup analysis was conducted. Previous research indicated that subgroup analyses should be interpreted with caution, we planned a priori to limit our subgroup analyses to a limited number of baseline characteristics including area, mean age, mean BMI, percentage of overweight/obese patients, percentage of primigravida, percentage of smoking patients, sample size, and quality score.

√	Assessment of study quality, including blinding of quality assessors; stratification or regression on possible predictors of study results	Two independent reviewers (RZL and YXH) used the established guidelines, Cochrane's "Tool to Assess Risk of Bias in Cohort Studies", to evaluate the methodological quality of the included studies, which has been widely used to evaluate observational studies.
√	Assessment of heterogeneity	Heterogeneity of the studies were explored within two types of study designs using Cochrane's Q test of heterogeneity and I^2 statistic that provides the relative amount of variance of the summary effect due to the between-study heterogeneity.
√	Description of statistical methods in sufficient detail to be replicated	Description of methods of meta-analyses, sensitivity analyses, meta-regression and assessment of publication bias are detailed in the methods.
√	Provision of appropriate tables and graphics	We included 1 flow chart,1 summary table, 1 forest plot of all studies, 1 funnel plot of publication bias, 1 table of subgroup analyses and 1 table of meta-regression analysis. In addition, we included 1 supplementary Figs and 3 supplementary tables in the supplementary data file.
**Reporting of results should include**		
√	Graph summarizing individual study estimates and overall estimate	Figure 2
√	Table giving descriptive information for each study included	Table 1
√	Results of sensitivity testing	Fig S1
√	Indication of statistical uncertainty of findings	95% confidence intervals were presented with all summary estimates, I^2 values and results of sensitivity analyses
**Reporting of discussion should include**		
√	Quantitative assessment of bias	The results of the Egger's test showed that publication bias was not found in this study and the sensitivity analysis showed that no individual study significantly influenced the overall results. However, the observed heterogeneity should be noticed.
√	Justification for exclusion	We excluded studies that not write in English or Chinese, which was a limitation in this review.
√	Assessment of quality of included studies	We discussed the results of the subgroup analyses, and potential reasons for the observed heterogeneity.
**Reporting of conclusions should include**		
√	Consideration of alternative	We noted that the variations in the incidence may be due to

	explanations for observed results	true population differences, or to differences in quality of studies, sample size, etc.
√	Generalization of the conclusions	Evidence suggests that the incidence of GDM were very common among PCOS patients. Further research is needed to explore more possible risk factors for GDM and identify effective strategies for preventing GDM among PCOS patients.
√	Guidelines for future research	During the process of screening data, we found that there were relatively few data on incidence of GDM among PCOS patients. Of the 22 included studies, 13 (59.09%) were rated as low quality and 59.09% of the included studies with a sample size ≤ 300. Thus, we think a large multicenter prospective study using a single validated measure of GDM and measuring possible confounding factors in randomly selected PCOS patients is needed in the future, which would provide a more accurate estimate of GDM among PCOS patients. Currently, the results of population-based studies of dietary or combined lifestyle measures have not indicated too much improvements in the risk of developing GDM. Besides, those trials involving physical activity programs have yielded conflicting results. Given the great potential for reducing the disease burden of PCOS patients, future research should continue to identify interventions that can be easily implemented in patients with PCOS, especially during their preconception period. Additionally, due to lack of data in many subgroups, we were unable to perform meta regression analysis for some possible confounders, such as socioeconomic status, family history of GDM, physical activity, drinking and diet habit. Thus, there might be a considerable amount of uncertainty regarding the pooled incidence of GDM among PCOS patients. Future research should, therefore, explore more potential risk factors for GDM among PCOS patients, especially genetic background as well as health-related behavior or other concomitant chronic diseases.
√	Disclosure of funding source	This research was supported by the Health Commission of Hunan Province (Grant NO: B2017167) and Hunan Pharmaceutical Association (Grant NO: Hn201707). The funding agency did not take part in the design of the study and collection, analysis, and interpretation of data and in writing the manuscript

Table S7

1	study (first author /publication	event	n	Area	country	income	criteria for GDN	quality	age	BMI	Overweigh	smoking	Primigravida	Irregular mense
2														
3														
4														
5														
6														
7														
8														
9														
10														
11														
12														
13														
14														
15														
16														
17														
18														
19														
20														
21														
22														
23														
24														
25														

Table S8 study characteristics of included studies

**First author**	**Diagnostic criteria for PCOS**
Sammeli West et al. (2020)	Self-report
Mahnaz Bahri Khomami et al. (2019)	self-report
J-Z Chen et al. (2016)	modified Rotterdam criteria
Ginevra Mills et al. (2020)	the 2003 Rotterdam criteria
S. Weerakiet et al. (2004)	the diagnosis of PCOS according to Homburg
Shiqiao Hu et al. (2021)	Patients with PCOS, who met the two-criteria for PCOS classification (oligo- or anovulation and polycystic ovary morphology), were assigned to the PCOS group
Hexia Xia et al. (2017)	the Rotterdam criteria
Dayan Liu et al. (2015)	not clear
Congcong Sun et al. (2019)	Chinese health industry criteria for PCOS
Xiangzun Li et al. (2017)	the 2003 Rotterdam criteria
Huizhuo Zhong et al. (2017)	the 2003 Rotterdam criteria
Marlieke deWilde et al. (2015)	the Rotterdam 2003 consensus criteria
R Helseth Vanky et al. (2013)	According to the Rotterdam criteria, PCOS diagnosis requires the presence of at least two of the three criteria
Guanghui Li et al. (2018)	the modified Rotterdam Criteria
V. De Fre`ne et al. (2014)	the Rotterdam criteria
Fatemeh Foroozanfard et al. (2020)	PCOS was present with at least two of the Rotterdam diagnostic criteria
R. Bond et al. (2017)	the Rotterdam criteria
Mahnaz Ashrafi et al. (2014)	presence of at least two of the Rotterdam criteria
Nadira Sultana Kakoly et al (2017)	self-report

Michael Feichtinger et al. (2021)	the Rotterdam criteria
M.A. deWilde et al. (2014)	the Rotterdam 2003 consensus criteria
Roos N et al. (2011)	ICD-10
